# Increased Oxidative and Nitrative Stress and Decreased Sex Steroid Relaxation in a Vitamin D-Deficient Hyperandrogenic Rodent Model—And a Validation of the Polycystic Ovary Syndrome Model

**DOI:** 10.3390/nu17020201

**Published:** 2025-01-07

**Authors:** Réka Eszter Sziva, Réka Kollarics, Éva Pál, Bálint Bányai, Ágnes Korsós-Novák, Zoltán Fontányi, Péter Magyar, Anita Süli, György L. Nádasy, Nándor Ács, Eszter Mária Horváth, Leila Hadjadj, Szabolcs Várbíró

**Affiliations:** 1 Department of Obstetrics and Gynaecology, Semmelweis University, Üllői Street 78/a, 1082 Budapest, Hungary; sziva.reka@semmelweis.hu (R.E.S.); fontanyi.zoltan@med.semmelweis-univ.hu (Z.F.); suli.anita@med.semmelweis-univ.hu (A.S.); acs.nandor@med.semmelweis-univ.hu (N.Á.); varbiro.szabolcs@semmelweis.hu (S.V.); 2 Department of Physiology, Semmelweis University, Tűzoltó Street 37–47, 1094 Budapest, Hungary; banyai.balint@phd.semmelweis.hu (B.B.); nadasy.gyorgy.laszlo@semmelweis.hu (G.L.N.); horvath.eszter.maria@semmelweis.hu (E.M.H.); 3 Workgroup of Research Management, Doctoral School, Semmelweis University, 1085 Budapest, Hungary; 4 Institute of Translational Medicine, Semmelweis University, Tűzoltó Street 37–47, 1094 Budapest, Hungary; pal.eva@med.semmelweis-univ.hu (É.P.); leila.hadjadj@gmail.com (L.H.); 5 Department of Obstetrics and Gynaecology, Szeged University, Semmelweis Street 1, 6725 Szeged, Hungary; 6 HUN-REN-SU Cerebrovascular and Neurocognitive Diseases Research Group, 1094 Budapest, Hungary; 7 Department of Pathology, Toldy Ferenc Hospital and Outpatient Clinic of Cegléd, Törteli Street 1–3, 2700 Cegléd, Hungary; novak.agnes@gmail.com; 8 Medical Imaging Centre, Faculty of Medicine, Semmelweis University, Üllői Street 78/a, 1083 Budapest, Hungary; drmagyarpeter@gmail.com

**Keywords:** female rodent model, vitamin D deficiency, hyperandrogenism, ovulatory dysfunction, estrogen, impaired relaxation, coronary artery, oxidative—nitrative stress, PCOS, rat model

## Abstract

Background/Objectives: Both hyperandrogenism (HA) and vitamin D deficiency (VDD) can separately lead to impaired vascular reactivity and ovulatory dysfunction in fertile females. The aim was to examine the early interactions of these states in a rat model of PCOS. Methods: Four-week-old adolescent female rats were divided into four groups: vitamin D (VD)-supplemented (*n* = 12); VD-supplemented and testosterone-treated (*n* = 12); VDD- (*n* = 11) and VDD-and-testosterone-treated (*n* = 11). Animals underwent transdermal testosterone treatment for 8 weeks. Target VD levels were achieved with oral VD supplementation and a VD-free diet. Estrous cycles were followed by vaginal smear, and quantitative histomorphometric measurements of the ovaries were also taken. In the 8th week, testosterone- and estrogen-induced relaxation of coronary arterioles was examined with pressure angiography. Estrogen receptor (ER) density and oxidative and nitrative stress parameters (Poly-(ADP-Ribose)-Polymerase and 3-nitrotyrosine) in the vessel wall were investigated with immunohistochemistry. Results: VDD caused impaired estrous cycles, and testosterone caused anovulatory cycles (the cycles were stopped at the diestrous phase). VDD combined with testosterone treatment resulted in reduced testosterone and estrogen vasorelaxation, lower ER density, and higher oxidative and nitrative stress in the vessel wall. Conclusions: PCOS with vitamin D deficiency may be associated with increased oxidative–nitrative stress in coronary arterioles. This oxidative and nitrative stress, potentially caused by hyperandrogenism and/or vitamin D deficiency, could impair estrogen-induced relaxation of the coronary arterioles, possibly by decreasing NO bioavailability and disrupting the estrogen-induced relaxation pathway.

## 1. Introduction

PCOS (polycystic ovary syndrome) is highly prevalent in all ethnicities all over the world; its global prevalence ranges from 10 to 13% [1]. PCOS is receiving more and more attention as it is one of the most prevalent causes of infertility. However, PCOS might have even more severe consequences affecting the health of women. PCOS can be interpreted as a multifactorial metabolic disease with gynecological manifestations. PCOS is associated with an increased risk of metabolic risk factors, diabetes, cardiovascular disease, and sleep disorders [2,3]. Rotterdam criteria serve as the basis of PCOS diagnosis. According to the 2023 International Evidence-based Guideline for the Assessment and Management of Polycystic Ovary Syndrome, the diagnostic criteria are the following in adults: the presence of two of (i) clinical/biochemical hyperandrogenism, (ii) ovulatory dysfunction, and (iii) polycystic ovaries on ultrasound or elevated Anti- Müllerian Hormone (AMH) levels, after other causes of these features were excluded [1].

It is known that PCOS increases cardiovascular risk. The data are mixed regarding the occurrence of cardiovascular events, such as myocardial infarction and stroke, in women with PCOS [4,5]. However, there is a consensus in the literature that PCOS is associated with higher cardiovascular risk [6,7]. There can be more factors which can contribute to the increased cardiovascular risk in PCOS, such as hyperandrogenism, insulin resistance, or diabetes.

Women with PCOS are also at increased risk of vitamin D deficiency (VDD). Several studies showed that VDD is a common co-morbidity with PCOS; ca. 67–85% of women with PCOS have vitamin D (VD) levels <20 ng/mL [8,9,10]. In recent years, several studies have suggested that vitamin D deficiency increases cardiovascular risk [11,12]. The data are controversial, but vitamin D deficiency seems to be a cardiovascular risk factor that can even contribute to adverse cardiovascular events [13,14].

To examine the combined vascular and ovarian effects of vitamin D deficiency and a hyperandrogenic state, a complex rodent model was created. In this model, the effects of hyperandrogenism and vitamin D deficiency on the estrous cycle, ovarian morphology, and the acute vasodilator effects of the sex steroids were examined.

The purpose of this study was to investigate the combined effect of hyperandrogenism and vitamin D deficiency on coronary arteries in a female PCOS rodent model. The initial steps of potential vascular damage were intended to be examined. Based on our previous studies, it is hypothesized that both pathological states could negatively affect vascular morphology and function and might increase oxidative–nitrative stress. We carried out our tests on the intramural branches of the left anterior descending artery, the main supplying vessel of the left ventricle in the rat.

## 2. Materials and Methods

### 2.1. Animals

Forty-six female Wistar rats, aged 4 weeks and averaging an initial body weight of 106 ± 7 g, were acquired from Semmelweis University—Charles Rivers in Budapest, Hungary. The rats were provided with tap water and rat chow ad libitum, following the specific protocols for their respective groups (details below). Initially, the rats were housed in groups of four to five, later reduced to two or three per cage, under controlled room temperature (22 ± 1 °C), constant humidity (56%), and a light–dark cycle of 12 h each. Blood sampling was conducted using superficial ether narcosis, while euthanasia was carried out through exsanguination following Nembutal anesthesia at a dosage of 45 mg/kg via intraperitoneal injection. This study conforms to the Guide for the Care and Use of Laboratory Animals published by the US National Institutes of Health [15] and the Hungarian Law on Animal Care (XXVIII/1998) conform the EU standards. The protocol was approved by the institutional Animal Care Commission (PEI/001/820-2/2015).

Abbreviations of the groups are as follows:

- VD+/T−: Vitamin D3-supplemented (T-naïve);
- VD+/T+: Vitamin D3-supplemented and transdermal testosterone (T)-treated;
- VD−/T−: Vitamin D3-deficient (T-naïve);
- VD−/T+: Vitamin D3-deficient and transdermal testosterone (T)-treated.

Significance symbols are labeled individually on each table and graph.

### 2.2. Chemicals

For the in vitro experiments, a standard Krebs–Ringer solution was utilized, composed of the following components (in mmol/L): NaCl 119, KCl 4.7, NaH_2_PO_4_ 1.2, MgSO_4_ 1.17, NaHCO_3_ 24, CaCl_2_ 2.5, glucose 5.5, and ethylene-diamine-tetraacetic acid (EDTA) 0.034. To induce relaxation in smooth muscle, a calcium-free Krebs solution was used, which included NaCl 92, KCl 4.7, NaH_2_PO_4_ 1.18, MgCl_2_ 20, MgSO_4_ 1.17, NaHCO_3_ 24, glucose 5.5, EDTA 0.025, and egtazic acid (EGTA) 2. The pH of both solutions was maintained at a stable level by keeping them at a constant temperature of 37 °C and continuously bubbling with a gas mixture consisting of 20% O_2_, 5% CO_2_, and 75% N_2_. 17-beta estradiol and the thromboxane A2 receptor agonist (U46619) were sourced from TOCRIS Bio-Techne (Bristol, UK), while testosterone was obtained from Sigma-Aldrich (Darmstadt, Germany). Transdermal testosterone gel (Androgel 1%) was acquired from Lab. Besins International S.A. (Paris, France). For oral vitamin D supplementation, cholecalciferol suspension (Vigantol oil 20,000 IU/mL from Merck/Merck Serono, Mumbai, India) was used. All chemicals were freshly prepared in a normal Krebs solution on the day of the experiment.

### 2.3. Chronic Treatment

This study lasted for 8 weeks for each group of rats, which were randomly assigned into four groups. Twenty-four rats were given a completely normal diet that included vitamin D at a concentration of 1000 IU/kg (SM Rat/mouse normal diet S8106-S011, Ssniff Spezialdiäten GmbH, Soest, Germany). Of these, 12 rats received transdermal testosterone treatment as described below (group VD+/T+, *n* = 12), while the other 12 rats formed the vitamin D-supplemented and testosterone-free group (VD+/T−, *n* = 12). To achieve optimal serum vitamin D levels (approximately 30 ng/mL of serum 25-hydroxycholecalciferol), additional oral vitamin D supplementation was administered (details below). Twenty-two rats were placed on a vitamin D-free diet (containing less than 5 IU/kg of vitamin D, EF Rat/mouse vitamin D-free diet E15312-24, Ssniff Spezialdiäten GmbH, Soest, Germany), ensuring significant vitamin D deficiency throughout this study. This group was divided into two halves: one half remained testosterone-free (group VD−/T−, *n* = 11), while the other half received transdermal testosterone treatment (group VD−/T+, *n* = 11). Body weight measurements were taken five times a week during the 8-week treatment period. Transdermal testosterone was administered at a dosage of 0.0333 mg/g five times weekly to induce hyperandrogenemia in the female rats. For this treatment, the backs of the treated animals were regularly shaved under sterile conditions. Vitamin D supplementation was calculated based on the normal dietary intake of vitamin D (1000 IU/kg), with the aim of achieving a total dietary vitamin D level of 3000 IU/kg to ensure elevated normal range levels. An oral loading dose was given in the 2nd week of treatment, followed by weekly oral administration of cholecalciferol starting from the 4th week. Blood samples were collected from the tail veins in the 4th and 8th weeks of treatment to assess levels of sex hormones and vitamin D.

Vitamin D supplementation resulted in serum 25-hydroxycholecalciferol levels that were approximately five times higher compared to the vitamin D-deficient groups. The reached serum 25-hydroxycholecalciferol levels after the 8th week of the treatment were (ng/mL; mean ± SEM) VD+T−: 32.328 ± 4.49; VD+/T+: 33.106 ± 4.46; VD−/T−: 6.044 ± 0.63; and VD−/T+: 6.006 ± 0.6. Further results of blood tests in this model have been reported previously [16]. Beginning in the 6th week, daily vaginal smear examinations were conducted to evaluate changes in the estrous cycle of the animals according to the different stages of the cycle. Throughout the 8-week treatment period, no medical or toxic complications were noted.

### 2.4. Pressure Angiography of the Coronary Arterioles and Calculation

In the 8th week of this study, the animals were anesthetized using Nembutal at a dosage of 45 mg/kg body weight (i.p., Ceva-Phylaxia, Budapest, Hungary). A cardiac ultrasound was conducted, and invasive blood pressure measurements were taken through cannulation of the carotid artery. Following thoracotomy to remove the heart, its weight was recorded, and the hearts were perfused with a non-heparinized normal Krebs solution (nKR) for 2 min. A segment of coronary arteriole with an in vivo outer diameter of 150–200 μm was isolated from the left anterior coronary artery by microdissection of the left ventricular muscle tissue under a stereomicroscope (Wild M3Z, Heerbrugg, Switzerland) [17]. The arteriole segment was then microcannulated in a heated organ chamber filled with a normal Krebs solution (Experimetria Ltd., Budapest, Hungary) and maintained at a constant temperature of 37 °C with oxygenation. The microcannulas were connected end-to-end to servo pumps (Living Systems, St. Albans Burlington, VT, USA). Under no-flow conditions, the coronary arteriole was intraluminally pressurized to 50 mmHg and extended to its normal in vivo length. The organ chamber featured a glass bottom to facilitate videomicroscopic microangiometry for recording changes in the arteriole segments. To assess the variations in outer and inner diameters (Do and Di) of the arterioles, this setup was aligned with the light path of a Leica microscope. Magnified images of the arteriole were captured using a Leica camera (Leica DFC 320, Leica Microsystems, Wetzlar, Germany) and analyzed with Leica QWin 3.4.0 image analysis software (Leica QWin, Leica Microsystems, Wetzlar, Germany). The vessel images were subsequently analyzed offline using both Leica QWin and FIJI—ImageJ software (ImageJ 1.52i, National Institutes of Health, Bethesda, MD, USA).

After allowing the arteriole to equilibrate in an oxygenated normal Krebs solution at a constant pressure of 50 mmHg and a temperature of 37 °C for 30 min, the steady-state diameter was measured. Pressure–diameter profile curves were generated by applying a training regimen (0–150–0–150 mmHg intraluminal pressure) and incrementally increasing the intraluminal pressure in 10 mmHg steps from 0 mmHg to 150 mmHg. At the end of a 10-min incubation at 50 mmHg, the resting diameter under this intraluminal pressure was reassessed. Subsequently, elevated doses of 17-beta estradiol (10^−8^ M, 10^−7^ M, 10^−6^ M, and 10^−5^ M) were added to the chamber. Each dose was incubated for 8 min, and then changes in vessel diameter were recorded. After this period, the drug was removed from the organ chamber by slowly flushing it with an oxygenated and heated normal Krebs solution. Following the washing period, the vessels were incubated for an additional 10 min. Next, two single doses of testosterone (10^−8^ M and 10^−6^ M) were added to the organ bath and incubated for 5 min. After this observation period, testosterone was washed out from the chamber, and the vessels were incubated for another 10 min in a normal Krebs solution. To assess the vasoconstrictive biomechanical properties of the vessels, a single high dose (10^−6^ M) of thromboxane A2 (TXA_2_) receptor agonist (U46619) was administered. Following a 5-min incubation, intraluminal pressure was gradually increased by 10 mmHg increments from 0 to 150 mmHg, while changes in both inner and outer diameters were recorded. Finally, after a resting period of 10 min at 50 mmHg, the diameters were measured again. The maximal smooth muscle relaxant potential of the vessel was determined by switching the organ chamber’s solution to a calcium-free Krebs solution, which was also heated and oxygenated. After a 20-min incubation period, the passive state of the vessel was evaluated by observing changes in inner and outer diameters in response to gradual increases in intraluminal pressure from 0 to 150 mmHg.

The following calculations were made: The basic biomechanical parameters, full vasoconstrictor and vasorelaxant capacities of the coronary arteries in response to different sexual steroids were computed for each dose of the given chemicals and each intraluminal pressure levels as follows:

- Inner radius (R_i_, μm):

(1) $$R_{i}=\frac{D_{i}}{2};$$

- Outer radius (R_o_, μm):

(2) $$R_{o}=\frac{D_{o}}{2};$$

- Full vasoconstriction (%):

(3) $$Full\;vasoconstriction\left(\%\right)=\frac{R_{Ca-free}-R_{TXA}}{R_{Ca-free}}\times 100;$$

- 17-beta-estradiol-induced vasorelaxation (%):

(4) $$E2-vasorelaxation\left(\%\right)=\frac{R_{E2}-R_{nKR}}{R_{nKR}}\times 100;$$

- Testosterone-induced vasorelaxation (%)

(5) $$T-vasorelaxation\left(\%\right)=\frac{R_{T}-R_{nKR}}{R_{nKR}}\times 100.$$

### 2.5. Vaginal Smear and Estrous Cycle

Starting in the 6th week of treatment, daily vaginal smear examinations were conducted to evaluate the estrous cycle of the animals. The samples were stained with a 1% methylene blue solution. Following microscopic analysis of the dominant cell types, estrous cycles lasting 4–5 days, which included at least three or four consecutive stages of the cycle, were considered normal. [18,19,20].

### 2.6. Ovarian Morphology and Quantitative Histomorphometry

At the termination of this study, the ovarian weight of each animal was recorded. Ovaries from individual animals in each group were collected for histological analysis and fixed in a 4% formaldehyde solution. Cross-sections of the ovaries, each 4 μm thick, were prepared. One set of slides was stained with hematoxylin–eosin (HE) to assess the histomorphometric parameters of the ovaries, while the remaining slides were used for immunohistochemical staining. After the digitalization of HE-stained slides, the total number, average diameter, and total area of follicles and corpora lutea (CL), as well as the total area of the ovary, were quantified using AxioVision Panoramic Viewer 4.7 software.

### 2.7. Immunohistochemistry of the Coronary Arterioles and Ovaries

Sections of native, paraffin-embedded ovarian tissue were stained for sexual steroids, specifically the beta subunit of the estrogen receptor and the androgen receptor, by BenchMark ULTRA Automated IHC/ISH slide staining system (Ventana Medical Systems, Inc., Tucson, AZ, USA) using monoclonal mouse anti-estrogen receptor beta antibodies (Santa Cruz Biotechnology, Dallas, TX, USA). Specific labeling was visualized using diaminobenzidine (DAB) as a brown substrate, with hematoxylin counterstaining providing a blue/violet color, achieved using the UltraView Universal DAB Detection Kit (Ventana Medical Systems, Inc.). Microscopic images of the stained vessels were captured using a Zeiss Axio Imager system (Zeiss, Oberkochen, Germany). The positively stained areas in the intimal and medial layers of the vessel walls were quantified as a percentage of the total tissue area using ImageJ software.

The poly-ADP-ribose (PAR) immunohistochemistry was conducted according to the manufacturer’s instructions [21]. Briefly, ovarian and coronary arteriolar samples were fixed in formalin, embedded in paraffin, and sectioned to a thickness of 5 μm. Following deparaffinization and antigen retrieval using a 0.1 mmol/L citrate buffer (pH 3) heated in a microwave for 15 min, the samples were incubated overnight at 4 °C with a mouse monoclonal anti-poly-ADP-ribose (PAR) antibody (Calbiochem, San Diego, CA, USA; dilution 1:1000). Secondary labeling was performed using a biotinylated anti-mouse horse antibody (Vector Laboratories, Burlingame, CA, USA) for 30 min at room temperature. This was followed by the application of horseradish peroxidase-conjugated avidin for another 30 min at room temperature and nickel-enhanced diaminobenzidine for 6 min at room temperature to produce a black color (Vector Laboratories). The tissue sections were then counterstained with nuclear fast red. To assess PAR staining, the following semiquantitative score system was used by a blinded observer as described previously [22,23].

Nitrative stress was characterized by 3-nitrotyrosine (NT) immunohistochemistry. It was performed following the manufacturer’s instructions as described previously [24]. Briefly, paraffin-embedded tissue sections of coronary arterioles were used for immunohistochemical analysis. After deparaffinization, antigen retrieval was achieved by heating the slides in citrate buffer (pH 3). Endogenous peroxidase activity was blocked with 3% H_2_O_2_ in distilled water, and nonspecific labeling was minimized using 2.5% normal horse serum (Vector Biolabs, Burlingame, CA, USA). The primary antibody, polyclonal rabbit anti-NT (1:500, Merck Millipore, Burlington, MA, USA), was applied overnight at 4 °C. Secondary labeling was conducted using horseradish peroxidase-linked anti-mouse monoclonal antibodies (Vector Biolabs), with visualization achieved through brown-colored diaminobenzidine (DAB, Vector Biolabs). Counterstaining was performed with blue hematoxylin (Vector Biolabs). Microscopic images of the tissue sections were captured using a Zeiss Axio Imager system (Zeiss, Oberkochen, Germany), and the uncalibrated optical density of the brown staining was estimated using ImageJ software (NIH, Bethesda, MA, USA). All assessments of immunohistochemical staining were conducted in a blinded manner.

### 2.8. Statistical Analysis

For statistical analysis, GraphPad Prism 6.0 (GraphPad Software, San Diego, CA, USA) was used. A two-way repeated-measures analysis of variance (ANOVA) was used for the statistical analysis of the curves (e.g., cumulative dose–response curves). Discrete parameters (e.g., body weight) were compared with one one-way ANOVA. Tukey test was used as a post hoc test, and *p* < 0.05 was uniformly accepted as the threshold for statistical significance. Missing data were handled as missing. Data are shown as mean ± SEM.

## 3. Results

### 3.1. Estrous Cycle Pattern and Ovary Weight

We have previously reported a significant difference in the final mean body weights of transdermal testosterone-treated animals compared to non-testosterone-treated ones. The efficacy of the treatment regime (VD and testosterone (T))—proved by the repeated measurement of serum vitamin D and sex hormone levels in the 4th and 8th weeks of treatment—was also published [16].

The number of normal estrous phases was identified according to the daily vaginal smear examination from the 6th week of treatment. The VD-supplemented T-free (‘double negative’ control) group presented normal, regular estrous cycles and had a significantly higher number of corpora lutea (i.e., higher ovulation rate) compared to the T-treated groups (Table 1, * *p* < 0.05). Vitamin D-deficient T-free rats had mostly irregular and significantly fewer estrous cycles compared to the double noxa-free group (Table 1, * *p* < 0.05). Testosterone-treated animals (regardless of VD status) showed anovulatory patterns (i.e., a significantly higher number of corpora lutea) and significantly fewer estrous cycles than transdermal T-naïve counterparts (Table 1, * *p* < 0.05 and ‡ *p* < 0.05).

After the 8th week of treatment, the ovarian weight was measured. Both T-treated groups, as well as the VD-deficient T-free animals, had a significantly lower ovarian weight compared to the double noxa-free group (Table 1, * *p* < 0.05).

### 3.2. Ovarian Morphology and Quantitative Histomorphometry

The total number of follicles, the number of corpus luteum, and the mean area of the follicles relative to the total ovarian area were calculated in a representative sample from each animal group (*n* = 6). In VD-supplemented, non-T-treated animals (VD+T−), dominant follicles and corpora lutea were detectable.

The mean number of detected follicles and their total ovarian area ratio was significantly higher in T-treated animals compared to their non-T-treated counterparts (VD+T−, VD−T−) (Table 1, * *p* < 0.05 and ^‡^ *p* < 0.01). These parameters were also significantly higher in VD-deficient animals compared to their VD-supplemented counterparts (Table 1, * *p* < 0.05 and ^†^ *p* < 0.05). A significantly lower number of corpora lutea was found in T-treated groups compared to their counterparts (Table 1, * *p* < 0.05 and ^‡^ *p* < 0.05). These changes were also presented in the corpus luteum area/total ovarian area ratio results (Table 1, * *p* < 0.05 and ^‡^ *p* < 0.05).

**Figure 1:**
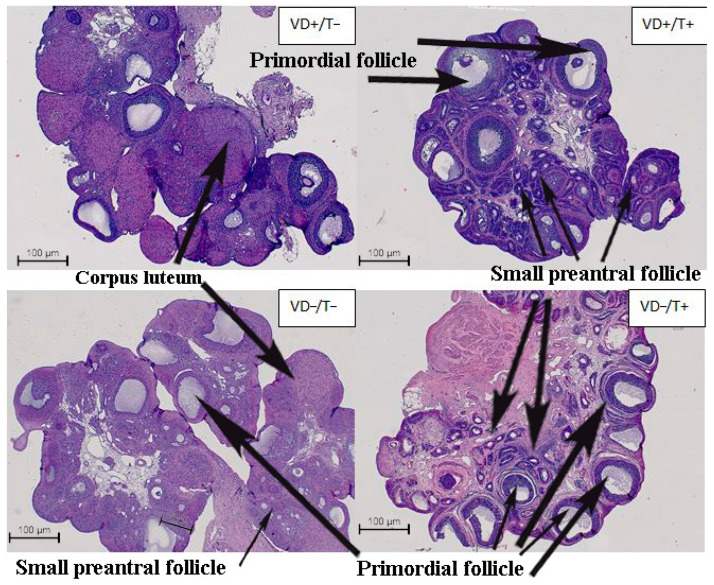
Representative hematoxylin–eosin-stained histological sections demonstrate the characteristic ovary morphology of each group. Only the VD+/T− animals showed normal follicle development and a normal number of corpora lutea. Polycystic ovary morphology was detectable after transdermal testosterone treatment. Interestingly, VD deficiency caused similar changes and led to an elevated number of non-dominant (primordial) follicles. VD+/T−: vitamin D3-supplemented; VD+/T+: vitamin D3-supplemented and transdermal testosterone-treated; VD−/T−: vitamin D3-deficient; and VD−/T+: vitamin D3-deficient and transdermal testosterone-treated.

Observing the microscopic (qualitative) ovarian morphology of vitamin D-treated and transdermal testosterone-naïve rats (VD+T−), dominant follicles and corpora lutea were detectable (Table 1). In the case of transdermal T-treated groups, a typical polycystic ovarian morphology was recorded (a higher number of follicles compared to the non-treated groups). In these groups, a large amount of small-sized primordial follicles and practically no or very few corpora lutea were detected (Table 1). Vitamin D deprivation (without T treatment) elevated the follicle numbers (Table 1) and resulted in a similar ovarian morphology. Figure 1 shows representative images of the hematoxylin–eosin-stained ovarian tissue sections with the above-mentioned typical changes.

### 3.3. Vascular Reactivity of the Coronary Arterioles

**Figure 2:**
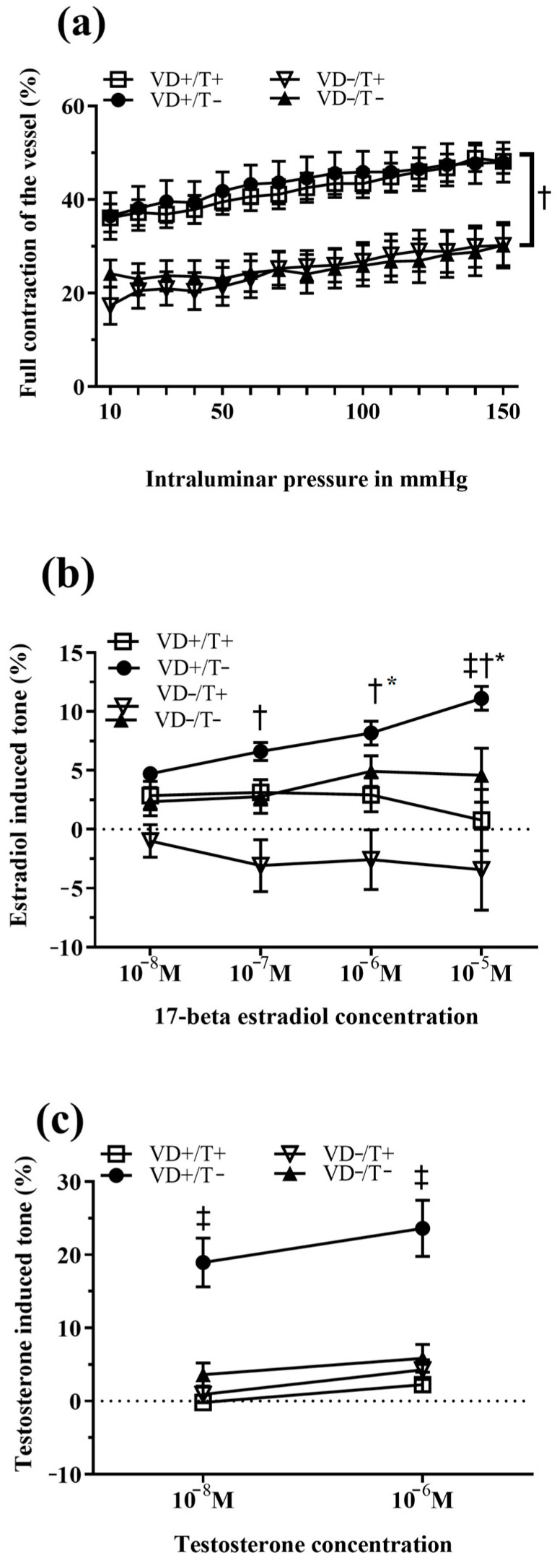
TXA_2_ agonist (U46619)-induced full contraction and sexual steroid-induced relaxation of the coronary arterioles (results are shown in mean ± SEM). (**a**) Inner radius in full contraction (after potent vasoconstrictive agent U46619) state as a function of intraluminal pressure. No difference in contractile capacity was observed between the two vitamin D-supplemented groups, both of which exhibited significantly higher values compared to the vitamin D-deficient groups (*p* < 0.05 in both comparisons). There was no difference in contractile capacity among the vitamin D-deficient groups. Both VD-deficient groups, regardless of transdermal T treatment, had significantly reduced contraction capacities compared to both VD-supplemented groups (†: *p* < 0.01, VD+/T− vs. VD−/T+ and VD+/T+ vs. VD−/T−). (**b**) Percent of vessel tone on fixed intraluminal pressure (50 mmHg) after administering an elevated dose of 17-beta estradiol. At higher 17-beta estradiol concentration values, the VD-deficient, transdermal T-treated group had significantly lower values compared to the VD-supplemented transdermal T-free group (†: *p* < 0.01 and *p* < 0.001 on elevated doses for VD−/T+ vs. VD+/T−). The VD-supplemented T-treated group showed similar concentration-dependent tone changes compared to the VD-supplemented non-T-treated group (*: *p* < 0.01 for VD+/T+ vs. VD+/T−). Significant tone reduction was detected among VD-deficient arterioles on the highest applied concentration (‡: *p* < 0.01 for VD−/T− vs. VD+/T−). (**c**) Percent of vessel tone on fixed intraluminal pressure (50 mmHg) after administering an elevated dose of testosterone. The VD-supplemented, transdermal T-free group showed significantly higher relaxation capacities compared to all other groups (‡: *p* < 0.001 on each dose for VD+/T− vs. VD+/T+, VD−/T−, and VD−/T+). Abbreviations: VD+/T−: vitamin D-supplemented; VD+/T+: vitamin D-supplemented and transdermal testosterone-treated; VD−/T−: vitamin D-deficient; VD−/T+: vitamin D-deficient and transdermal testosterone-treated; and M: drug concentration in mol/L applied in the organ chamber. *n* = 11 in all groups. The significance of the two-way ANOVA tests is shown. All values are expressed in mean ± SEM.

We have already reported that VD deficiency, regardless of T treatment, resulted in severe biomechanical alterations in the vessels’ geometry and relaxation capacities. Full contraction of the vessels was studied under elevated intraluminal pressure conditions. Both VD-deficient groups showed significantly reduced contraction capacities compared to the supplemented ones (Figure 2a, †: *p* < 0.01 in both comparisons VD+/T− vs. VD−/T+ and VD+/T+ vs. VD−/T−). A significant reduction in 17-beta estradiol relaxation capacities was found in the VD-deficient transdermal T-treated group compared to the VD-supplemented transdermal T-free groups (Figure 2b, †: *p* < 0.01 and *p* < 0.001). Interestingly, this difference was more pronounced when the concentration of the added estradiol was elevated and turned into vasoconstriction at the highest doses (negative relaxation). A similar, significant tendency was observed in the VD-supplemented, T-treated group (*: *p* < 0.05 and *p* < 0.01) and in the VD-deficient, non-T-treated group at the highest concentration (‡: *p* < 0.05). Testosterone-induced relaxation was only preserved in the VD-supplemented, transdermal testosterone-free arterioles. The other three groups had significantly lower relaxation values (Figure 2c, ‡: *p* < 0.001 in all comparisons).

### 3.4. Immunohistochemical Reactivity of the Coronary Arterioles and the Ovaries

**Figure 3:**
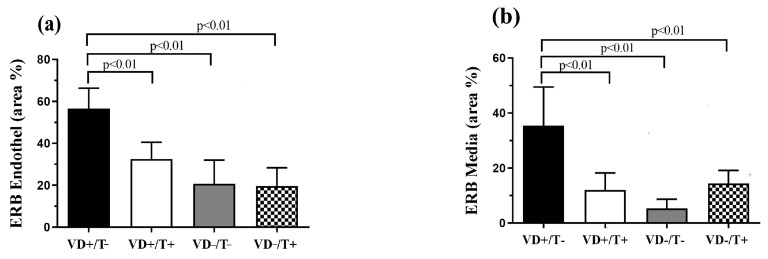
Immunohistochemical examination of estrogen receptor beta (ERB) specific immunostaining positivity across the vessel wall. Positively stained tissue area compared to total tissue area of the endothelial (**a**) and media layers (**b**). A significantly lower estrogen receptor density was observed in all examined layers if any of the two noxae were presented (*p* < 0.01 in all comparisons). The values are in total area ratio percentages; *n* = 6 in all groups. VD+/T−: vitamin D-supplemented; VD+/T+: vitamin D-supplemented and transdermal testosterone-treated; VD−/T−: vitamin D-deficient; VD−/T+: vitamin D-deficient and transdermal testosterone-treated; and ERB: estrogen receptor beta. The significance of the one-way ANOVA tests is shown above the bars. All values are expressed in mean ± SEM.

Estrogen receptor-specific immunostaining positivity of the arteriolar wall was significantly higher in VD-supplemented, transdermal T-free animals compared to all other groups. Overall, this characteristic of receptor distribution was true in the endothelial and media layer (Figure 3a,b, *p* < 0.01 in all comparisons: VD+/T− vs. VD+/T+, VD−/T−, and VD−/T+).

**Figure 4:**
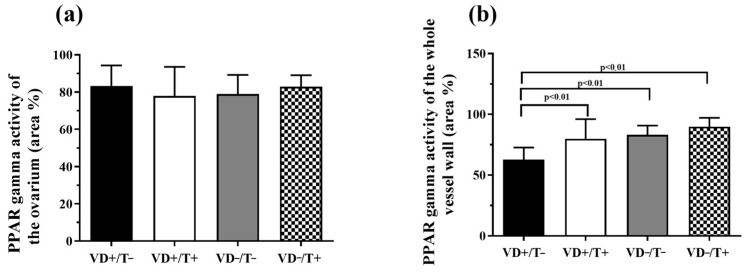
Poly(ADP-ribose) (PAR) formation in the ovarian tissue (**a**) and the vessel wall (**b**). In the ovary samples, no significant difference in the PAR-positive nuclear area ratio was detected between the four groups. The coronary arterioles of the VD-supplemented transdermal T-free animals had the lowest detected PARP activity (*p* < 0.01 in all comparisons). The values are in total nuclear area ratio percentages; *n* = 6 in all groups. VD+/T−: vitamin D-supplemented; VD+/T+: vitamin D-supplemented and transdermal testosterone-treated; VD−/T−: vitamin D-deficient; and VD−/T+: vitamin D-deficient and transdermal testosterone-treated. The significance of the one-way ANOVA tests is shown above the bars. All values are expressed in mean ± SEM.

Poly-(ADP-Ribose)-polymerase (PARP) activity was estimated by PAR immunohistochemistry in ovaries and coronary arterioles (Figure 4a,b). In the ovarian tissue, we detected no significant difference between the four groups (Figure 4a). Regarding the coronary arterioles, PARP activity of the double noxa-free group (VD+/T−) was significantly lower than any of the other groups (Figure 4b, *p* < 0.01 in all comparisons: VD+/T− vs. VD+/T+, VD−/T−, and VD−/T+).

**Figure 5:**
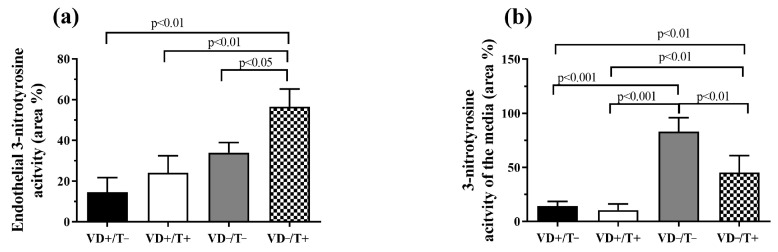
Expression of 3-nitrotyrosine formation in the vessel wall: (**a**) endothelium, (**b**) media layer. Regarding endothelial activity, the VD-deficient, T-treated group showed a significant increase in 3-nitrotyrosine-specific immunostaining positivity compared to the other groups *(p* < 0.01 and *p* < 0.05, respectively). In the media layer, the VD-deficient, T-free animals had significantly higher values than all other groups *(p* < 0.01 and *p* < 0.001); however, the VD-deficient, T-treated animals had significantly increased staining compared to both VD-supplemented groups (*p* < 0.01). The values are in total area ratio percentages; *n* = 6 in all groups. VD+/T−: vitamin D-supplemented; VD+/T+: vitamin D-supplemented and transdermal testosterone-treated; VD−/T−: vitamin D-deficient; and VD−/T+: vitamin D-deficient and transdermal testosterone-treated. The significance of the one-way ANOVA tests is shown above the bars. All values are expressed in mean ± SEM.

3-nitrotyrosine-specific immunostaining intensity of the endothelial layer was significantly higher in the double noxa group (Figure 5a, *p* < 0.01 compared to both VD-supplemented groups: VD−/T+ vs. VD+/T− and VD−/T+ vs. VD+/T+ and *p* < 0.05 VD−/T+ vs. VD−/T−). In the media layer, VD-deficient, T-free vessels had the highest activity (Figure 5b, *p* < 0.01 VD−/T− vs. VD−/T+, and *p* < 0.001 for VD−/T− vs. VD+/T− and VD+/T−); however, VD-deficient T-treated ones also differed significantly from both VD-supplemented groups (Figure 5b, *p* < 0.01 VD−/T+ vs. VD+/T+ and VD+/T−).

## 4. Discussion

Vitamin D insufficiency and female hyperandrogenism are frequent co-morbidities affecting 7–12% of women worldwide. The aim of the current study was to investigate the possible interplay between these two endocrinopathies, with special regard to the direct effects of sexual steroids on coronary arterioles. We have already reported the basic biomechanical changes in the coronary vessels: significantly thicker vessel wall, smaller inner lumina, reduced myogenic tone, and less response to increasing concentrations of local vasodilator adenosine were all the characteristics of vitamin D-deficient arterioles [25]. Interestingly, changes were only partially restored by vitamin D supplementation, which led us to the conclusion that both noxae could induce the remodeling of the coronary arterioles.

In the current model, testosterone-treated animals showed anovulatory patterns (Table 1 shows there was a significant decrease in the number of the corpora lutea) and significantly fewer estrous cycles than transdermal T-naïve counterparts (Table 1). Moreover, all testosterone-treated animals showed polycystic ovarian morphology. With these results, the current polycystic ovary syndrome rodent model was validated. It is already known that high testosterone excess causes polycystic ovary (PCO) morphology in rodent models (both dihidro-testosterone (DHT) and letrozole—an aromatase inhibitor treatment) [26,27,28]. Van Houten et al. created a PCO model in mice where the animals were treated with a 90-day continuous-release pellet containing the nonaromatizable androgen DHT or vehicle. In their model, they also observed that all DHT-treated mice were acyclic, their ovaries were devoid of corpora lutea, and the animals had anovulatory cycles [29]. Sucquart et al. developed a rodent model where the effects of prenatal androgen excess (PNA-modeling lean PCOS) and prepubertal androgen excess (PPA-modeling obese PCOS) were described. Consistent with the literature, in their study, both PPA and PNA mice were acyclic, indicating ovulatory dysfunction. However, a decreased number of corpora lutea was observed only in the PPA group [30]. Bakhshalizadeh et al. created a PCOS mouse model using a 20-day dehydroepiandrosterone (DHEA) injection regimen. Polycystic ovaries and absent corpora lutea were observed [31].

In the present study, the aim was to model PCOS, which is the most frequent cause of anovulation. This study described that not only did the well-known high androgen effect lead to PCO morphology, but vitamin D deficiency can also result in polycystic ovarian morphology. Moreover, vitamin D deficiency with testosterone treatment resulted in a lower number of corpora lutea (anovulatory cycles) and significantly fewer estrous cycles than the transdermal T-naïve counterparts.

Whereas the effect of chronic high androgen excess in the ovaries is well-characterized in rodent models, the effect of vitamin D deficiency on the ovaries is rarely examined. Vitamin D is supposed to have an important role in female reproduction [32,33]. Data from animal models and human studies are also consistent with this statement. However, the mechanisms by which vitamin D deficiency adversely affects fertility are not fully understood yet [32,33,34,35,36,37].

Dicken et al. examined young adult female rats maintained on a vitamin D-deficient diet after puberty. In the vitamin D-deficient Cyp27b1 (the rate-limiting enzyme in the synthesis of 1.25-dihydroxyvitamin D_3_) null group, the follicular development had been arrested, and the prolonged estrous cycles were characterized by extended periods of diestrous. Normal estrous cycles could be restored with vitamin D supplementation. Vitamin D-deficient, Cyp27b1 null mice also exhibited irregular estrous cycles, which can be reversed after a 4-week-long vitamin D supplementation. The ovaries collected from the vitamin D-deficient Cyp27b1 null groups showed reduced follicular heterogeneity with a lower number of corpora lutea. Moreover, most follicles in the ovaries of this group were arrested in the preantral stage [38]. These findings are consistent with the current results described here, as irregular and significantly longer estrous cycles were observed in the vitamin D-deficient groups in this study. Furthermore, a significantly higher number of follicles (polycystic ovary morphology) was detected in vitamin D-deficient animals, which has not been described in a rodent model so far. In the case of vitamin D deficiency, there can be multiple factors and hormonal abnormalities which might lead to abnormal follicular development and, as a result, polycystic ovary morphology [39].

Vitamin D insufficiency and female hyperandrogenism are frequent co-morbidities in humans. In the current rodent model, this hormonal and metabolic status was created in the vitamin D-deficient, testosterone-treated ‘double noxa’ group. In this group, the mean number of the estrous cycles was lower compared to the vitamin D-supplemented groups. Moreover, the mean number of follicles was significantly higher in this group. According to these results, vitamin D deficiency deteriorates the effects of hyperandrogenic status. Human studies have shown that lower vitamin D levels correlate with worse PCOS symptoms and clinical parameters (e.g., fasting glucose, fasting insulin levels, triglycerides, and HDL levels) [9,11].

However, the pathomechanism of this effect is not yet fully understood. Some studies examined the link between vitamin D levels and the gonadal production of androgens in fertile women; however, the results are controversial. In a prospective study of 100 non-obese and obese women with PCOS, it was shown that 25-hydroxy-vitamin-D3 (25-OH-D3) levels were negatively correlated with total testosterone levels [40]. In contrast, Parikh et al. observed in vitro that 25-OH-D3 had no significant effect on testosterone production in human ovarian cells [41]. This observation could suggest that decreased vitamin D levels might correlate with androgen levels due to systemic rather than intracellular regulation. Moreover, Chang et al. found that vitamin 25-OH-D3 levels positively correlate with total and free testosterone levels but not with dehydroepiandrosterone-sulphate (DHEA-S) or other steroid hormones in 73 fertile healthy women [42]. In summary, further studies are needed to investigate the exact effect of vitamin D on steroidogenesis.

On a molecular level, it is observed that vitamin D deficiency can lead to elevated androgen levels through different pathways. Hong et al. observed in vitro that vitamin D increases the expression of the aromatase CYP19A1 enzyme, which converts androgens to estrogen, in porcine ovarian granulosa cells [43]. In contrast, Bakhshalizadeh et al. observed an increased expression of CYP19A1 in their PCOS mouse model. Their PCOS model was created by a 20-day-long DHEA injection treatment. Granulosa cells were isolated and cultured with and without vitamin D. A significant decrease in the CYP19A1 expression was observed after the treatment with vitamin D [31]. This difference might have been caused by the different species and different models—it might have been a direct effect of agents used to provoke PCOS. However, it raises the question of what might occur in human conditions.

There are several human interventional studies on the effects of vitamin D supplementation on androgen levels; however, the results are conflicting [44].

Karadağ et al. observed that vitamin D supplementation decreased androgen levels in vitamin D-deficient women with PCOS [45]. On the contrary, other human interventional studies have found no change in androgen levels after vitamin D supplementation [46,47]. More large-scale interventional human studies are needed to evaluate the effects of vitamin D supplementation on androgen levels.

Everything considered, vitamin D deficiency might be another factor that decreases fertility, and together with PCOS, it may exacerbate the effects of the condition.

Hyperandrogenic status and vitamin D deficiency might have effects not only on fertility but also on cardiovascular health. It is widely known that female hormonal status strongly correlates with cardiovascular health and cardiovascular risks [48]. It is also known that women with PCOS have a higher cardiovascular risk [1], which might be further elevated by vitamin D deficiency [11,14,49]. Inspired by these previous reports, this study aimed to examine the initial vascular effects of these hormonal states, focusing on the coronary arteries in a rodent model.

Full contraction of the coronary segment was counted as a relative difference in maximal contraction and relaxation (full relaxation reached via Ca-free and contraction via TXA2). Both vitamin D-deficient groups had a significantly reduced vascular adaptation range (contraction and relaxation) compared to the vitamin D-supplemented groups. Thus, in the case of vitamin D supplementation, the adaptation range remained intact, even in the testosterone-treated groups. A lower adaptation range might result in vulnerable coronary arteries during stress situations. Altered sexual steroid-dependent relaxation was observed in PCOS earlier [21]. To understand the underlying mechanisms of this phenomenon, sexual steroid-dependent vasodilation and oxidative–nitrative stress were investigated in this study.

After administrating elevated doses of 17-beta estradiol, the relaxation decreased in all groups compared to the vitamin D-supplemented, testosterone-free ‘control’ group. One noxa (vitamin D deficiency or hyperandrogenic status) resulted in a decreased relaxation capacity, but the double noxa led to negative relaxation (contraction) at the highest dose (supraphysiological dose). The receptor density was also examined. A significantly lower estrogen receptor density was observed in all examined layers as well as throughout the entire vessel wall when either of the two noxae was present (Figure 3). However, an additional decrease in expression did not occur with the presence of both noxae. Increased oxidative–nitrosative stress may be a contributing factor in this group.

It is well-known that estradiol has direct (non-genomial) and indirect (genomial) vasodilator effects [50,51,52,53]. Estradiol has estrogen membrane receptors (ER) in the human vascular endothelium, which convey the direct, non-genomial vascular estrogen effects [54]. A subpopulation of ER-alpha (ERα) is present in caveolae in endothelial cells. Estrogen binding to ERα leads to G-protein-coupled receptor 30 (GPR30) activation. The downstream signaling leads to endothelial nitric oxide synthase (eNOS) activation within minutes, which results in the transient production of nitric oxide (NO) and, consequently, vasodilation [55,56,57].

The genomial estrogen effects are mediated by nuclear ER (ERα and ERβ) and can take more hours to manifest. In this way, estrogen can regulate gene transcription; for example, it upregulates eNOS and downregulates NADPH oxidase (NOX), so it has an indirect antioxidant effect. This process leads to a decrease in oxidative stress and helps preserve and produce more NO, thereby supporting vasorelaxation.

In the current study, the non-genomial direct vascular effects of estrogen were examined. Reduced capacity for relaxation and estrogen receptor density were observed in all vitamin D-deficient groups compared to the vitamin D-supplemented, testosterone-untreated ‘control’ group. In our previous studies, it was also detected that chronic vitamin D deficiency had a negative effect on estrogen-dependent vasorelaxation in the thoracic aorta of rats [58].

Similar to estrogen, vitamin D also has genomial and non-genomial effects. Vitamin D can reduce reactive oxidative and nitrosative species (ROS and RNS) in several ways, thereby increasing the bioavailability of NO, which is known to play an important role in vasodilation and is crucial in the downstream signaling pathway of the estrogen vascular effect [57,59]. Therefore, in vitamin D deficiency, these beneficial effects are absent, and the antioxidant effects of vitamin D are lacking.

Significantly increased PAR staining was observed in all vitamin D-deficient groups compared to the vitamin D-supplemented, testosterone-naïve ‘control’ group as a marker of elevated oxidative stress. In the vitamin D-supplemented, testosterone-treated group, PAR density was also significantly higher than in the control group.

In summary, vitamin D deficiency and a hyperandrogenic state can also cause increased oxidative stress—they can potentiate each other’s effects. The presence of both noxae (vitamin D deficiency and a hyperandrogenic state) resulted in increased 3-NT staining, indicating elevated nitrative stress. The increased oxidative and nitrative stress might contribute to the impaired estrogen-dependent relaxation capacity in vitamin D-deficient or hyperandrogenic groups. In this case, the bioavailability of NO decreases due to reactive oxygen species (ROS) and reactive nitrogen species (RNS), which can lead to impaired vasorelaxation ability. According to the aforementioned results, oxidative and nitrative stress is already present in the early stages of hyperandrogenism and vitamin D deficiency. This suggests that even a short duration (8 weeks) is sufficient for vascular damage to develop.

Besides the increased oxidative and nitrative stress, another important factor is the decreased estrogen receptor density, which can contribute to impaired vasorelaxation in vitamin D-deficient and/or testosterone-treated groups. Vitamin D and estrogen interact with each other. Vitamin D increases the ER expression in vascular tissues, and estrogen increases vitamin D receptor (VDR) expression in vascular smooth muscle. It is also possible that they may amplify each other’s vascular protective effects. Thus, vitamin D deficiency might not only lead to a loss of the beneficial vascular effects of vitamin D but also reduce the beneficial vascular effects of estrogen.

The current study also examined the direct (acute) effect of testosterone on vascular reactivity. Testosterone-induced relaxation was only preserved in the arterioles of the vitamin D-supplemented and testosterone-untreated group. The other three groups had significantly lower relaxation. In the vitamin D-supplemented testosterone-free group, the direct vasorelaxant effect of the circulating testosterone might have had cardiovascular benefits. The cardiovascular effects of testosterone are an actively researched, controversial issue [60,61]. Several studies have described that testosterone may be cardioprotective in men on a physiological level [62,63,64]. However, physiological testosterone levels may have similar effects in women as well. Several animal studies have also observed the vasodilatory effect of testosterone, consistent with the results described in this study [65,66,67,68]. Yue et al. detected testosterone-induced endothelium-independent vasodilatation in isolated rabbit coronary arteries and aorta [69]. Deenadayalu et al. described the potential signal pathway of testosterone and estrogen in the coronary arteries of male and female porcines(pigs) [70]. Their results show that in the vascular smooth muscle cell, testosterone and estrogen exert their effect through the PI3 kinase-Akt/NO/cGMP/PKG signaling cascade. This cascade mediates the acute, non-genomic, endothelium-independent relaxation effect of testosterone, which leads to the stimulation of large conductance voltage- and calcium-gated potassium channels. This causes the repolarization of vascular smooth muscle cells and leads to arterial relaxation [70].

NO plays an important role in this pathway. It can derive from two sources: endothelium and vascular smooth muscle [67,71,72]. In the case of oxidative stress, reactive oxygen species are generated, which react with NO, and consequently, the NO bioavailability decreases [73,74]. This may be a mechanism underlying impaired vasorelaxation in vitamin D deficiency or in a chronic hyperandrogenic state. It is known that both vitamin D deficiency and a hyperandrogenic state (PCOS) can lead to increased oxidative stress in vascular smooth muscle [75,76,77,78]. Chignalia et al. showed that testosterone induces ROS generation in a time- and concentration-dependent manner in the vascular smooth muscle cells (VMSC) of male Wistar–Kyoto normotensive rats. Only 8-h-long testosterone treatment resulted in augmented NOX1 expression (the enzyme which has a key role in ROS production) [79]. A chronic hyperandrogenic state also leads to increased ROS production, which explains why hyperandrogenism might cause altered vascular reactivity by disrupting the PI3 kinase-Akt/NO/cGMP/PKG signaling cascade.

Consistently, significantly higher PAR and 3-NT expression were detected in the testosterone-treated and/or vitamin D-deficient groups compared to the vitamin D-supplemented, testosterone-untreated ‘control’ group. Thus, increased oxidative and nitrative stress were present in these groups, which could decrease NO bioavailability and contribute to impaired vasorelaxation in vitamin D deficient and/or testosterone-treated groups.

According to our results, there is a decrease in both vasoconstrictor and vasodilator responses in vitamin D-deficient and/or hyperandrogenic groups. In this way, the adaptational range of the coronary arteries is narrowed, which causes reduced coronary perfusion and worsens the blood supply of the left ventricle of the heart.

Strengths and limitations: To the best of our knowledge, this is the first study investigating the vasoconstrictor and vasodilator responses of coronary arterioles in vitamin D-deficient and/or hyperandrogenic female rat groups. Moreover, this study is the first to describe polycystic ovary morphology in a vitamin D-deficient female rodent model. There are some limitations to this study: the small sample size and the absence of a naive animal group receiving a normal diet without any additional vitamin D supplementation. However, this study aimed to compare clearly deficient and optimal vitamin D serum levels; the optimal vitamin D level was reached successfully in the vitamin D-supplemented group. To estimate the sample size, a historical power analysis was conducted.

Further basic, translational, and clinical animal and human studies are needed to investigate the effects of vitamin D deficiency and hyperandrogenism on the functional and histological properties of coronary arterioles and to evaluate the potential vascular protective effects of vitamin D.

## 5. Conclusions

In conclusion, a hyperandrogenic state accompanied by vitamin D deficiency in females might result in higher oxidative and nitrative stress in coronary arterioles. Under these conditions, the narrowing of the vascular adaptation range and the increase in oxidative–nitrosative stress may occur even in individuals with normal blood pressure. These might be the earliest alterations in developing vascular damage. The oxidative and nitrative stress, potentially caused by hyperandrogenism and/or vitamin D deficiency, could impair estrogen-induced relaxation of the coronary arteries, possibly by decreasing NO bioavailability and disrupting the estrogen-induced relaxation pathway.

## Figures and Tables

**Table 1:** Ovarian function and morphology in Vitamin D-treated and/or testosterone-treated and untreated groups: estrous cycles, ovary weight, follicle count, number of corpora lutea, and area ratios (all results are shown in mean ± SEM; significances of one-way ANOVA tests are shown).

	VD+/T−	VD+/T+	VD−/T−	VD−/T+
Mean number of estrous cycles (6–8th week of treatment) ^1^	3.63 ± 0.15	0.91 ± 0.21 *	2.00 ± 0.23 *^,†^	0.36 ± 0.20 *^,‡^
Final mean ovarian weight (grams) ^1^	0.25 ± 0.09	0.06 ± 0.01 *	0.07 ± 0.01 *	0.068 ± 0.02 *
Mean number of follicles (pieces) ^2^	13.66 ± 1.72	58.50 ± 1.94 *	35.01 ± 2.68 *^,†^	71.33 ± 5.76 *^,‡,†^
Follicle area/total ovarian area ratio × 100 (%) ^2^	19.27 ± 1.29	43.99 ± 1.17 *	29.02 ± 4.49 *^,†^	38.29 ± 2.56 *
Mean number of corpora lutea (pieces) ^2^	12.67 ± 1.85	0.83 ± 0.40 *	5.16 ± 1.22 *^,†^	1.16 ± 0.40 *^,‡^
Corpus luteum area/total ovarian area ratio × 100 (%) ^2^	48.12 ± 3.19	0.68 ± 0.26 *	11.56 ± 3.26 *^,†^	0.82 ± 0.27 *^,‡^

^1^ *n* = 11/groups; ^2^ *n* = 6/groups. Abbreviations: VD+/T−: vitamin D-supplemented, transdermal testosterone-free; VD+/T+: vitamin D-supplemented and transdermal testosterone-treated; VD−/T−: vitamin D-deficient, transdermal testosterone-free; and VD−/T+: vitamin D-deficient and transdermal testosterone-treated. Significance symbols and *p*-values: * → significant difference compared to VD+/T− group: *p* < 0.05; ^†^ → significant difference compared to VD+/T+ group: *p* < 0.05; ^‡^ → significant difference compared to VD−/T− group: *p* < 0.05.

## Data Availability

The published article includes all data generated and analyzed from this series.

## List of Abbreviations

25-OH-D3	25-hydroxy-vitamin-D3
Akt	Protein kinase B
AMH	Anti-Müllerian Hormone
ANOVA	Analysis of variance
cGMP	Cyclic Guanosine Monophosphate
CL	Corpus Luteum
DAB	Diamino-Benzidine
DHEA	Dehydroepiandrosterone
DHEA-S	Dehydroepiandrosterone-sulphate
DHT	Dihidro-testosterone
eNOS	endothelial nitric oxide synthase
ER	Estrogen Receptor
GPR30	G-protein-coupled receptor 30
HA	Hyperandrogenism
HDL	High-Density Lipoprotein
HE	Hematoxylin–eosin
NADPH	Nicotinamide Adenine Dinucleotide Phosphate
NO	Nitric Oxide
NOX	NADPH oxidase
NT	3-nitrotyrosine
PAR	Poly-ADP-ribose
PARP	Poly-(ADP-Ribose)-polymerase
PCO	Polycystic ovary
PCOS	Polycystic Ovary Syndrome
PI3 Kinase	Phosphoinositide 3-kinase
PKG	Protein Kinase G
PNA	Prenatal androgen excess
PPA	Prepubertal androgen excess
RNS	Reactive Nitrogen Species
ROS	Reactive Oxygen Species
T	Testosterone
TXA2	Thromboxane-A2
VD−/T−	Vitamin D3-deficient and T-naïve
VD−/T+	Vitamin D3-deficient and transdermal testosterone (T)-treated
VD	Vitamin D
VD+/T−	Vitamin D3-supplemented and T-naïve
VD+/T+	Vitamin D3-supplemented and transdermal testosterone (T)-treated
VDD	Vitamin D Deficiency
VDR	Vitamin D Receptor
VMSC	Vascular smooth muscle cells
